# Factors influencing quality of life in patients with temporal lobe epilepsy

**DOI:** 10.1055/s-0045-1802552

**Published:** 2025-03-03

**Authors:** Amina Tani, Nawal Adali

**Affiliations:** 1 Ibn Zohr University, National School of Applied Sciences, Health Sciences Department, Agadir, Morocco.; 2 Ibn Zohr University, Faculty of Medicine and Pharmacy, REGNE Research Laboratory, Agadir, Morocco.; 3 University Hospital of Agadir, Department of Neurology, Morocco.

**Keywords:** Epilepsy, Temporal Lobe, Quality of Life, Cognition, Depression, Anxiety

## Abstract

**Background** Temporal lobe epilepsy (TLE) predisposes individuals to cognitive difficulties and psychosocial consequences. Evaluating its impact on quality of life (QOL) is essential for patient care.

**Objective** To identify factors influencing QOL in low-income patients with TLE.

**Methods** An institution-based cross-sectional study was conducted on 40 patients with TLE during neurological consultations at a day clinic in Agadir, Morocco. The Quality of Life in Epilepsy Inventory-31 (QOLIE-31) was used to measure QOL. Multivariate linear regression analysis was performed to assess the associations between QOL and demographic, clinical, psychiatric, social, and cognitive variables. Results were considered statistically significant at a *p* -value < 0.05.

**Results** The mean overall QOL score was 48.14 ± 22.02. Among the seven scales of the QOLIE-31, the Seizure Worry scale had the lowest mean score. Cognitive function, social support, and self-esteem were positively associated with QOL. In contrast, memory complaints, seizure duration, seizure frequency, anxiety, and depression were negatively associated with QOL.

**Conclusion** While current interventions primarily target seizure control, our findings emphasize the need for holistic approaches that address both cognitive and psychosocial challenges to optimize QOL.

## INTRODUCTION

Epilepsy is a major neurological disorder affecting ∼ 0.5 to 1% of the world's population. 1 Its complex implications across physical, mental, and social domains 2 contribute to a significant public health burden. This burden is particularly acute in developing countries, which are home to over 80% of the global cohort of people with epilepsy. 3 These regions are experiencing increased epilepsy-related mortality, often due to untreated manifestations, compounded by stigma and gaps in diagnostic and therapeutic infrastructure. 4

This concern is heightened in the context of temporal lobe epilepsy (TLE), a subset often resistant to antiseizure medications (ASMs), with hippocampal sclerosis being the most common cause. 5 6 Despite the efficacy of surgical interventions in reducing seizures, their adoption remains limited in resource-poor countries. Patients with TLE, particularly when untreated, suffer from cognitive impairment, anxiety, depression, low self-esteem, and social barriers. 7 8

Quality of life (QoL) is a key measure to understand the experiences of patients with epilepsy (PWEs) living in developing countries. 9 The literature 10 11 12 13 indicates that QoL in epilepsy patients is influenced by biomedical and psychosocial factors, including seizure frequency, severity, chronicity, anxiety, depression, low self-esteem, adverse effects of ASMs, social isolation, and lack of social support. The role of cognitive functioning in QoL remains under debate, with studies 14 15 16 17 showing mixed results.

There is a notable lack of research on QoL among PWEs living in developing countries, which makes it challenging to understand global QoL variations. 9 18 19

The present study investigates the determinants of QoL in economically-disadvantaged patients with TLE living in a region of Morocco. These patients often face limited access to optimal care due to sociocultural barriers, a shortage of neurologists, and inadequate medical treatment and follow-up. 4 20

The research aims to identify the clinical, demographic, psychological, social, cognitive, and therapeutic factors influencing the QoL in economically-disadvantaged patients with TLE. The findings are expected to guide healthcare professionals in improving prevention, therapy, and rehabilitation practices for this population.

## METHODS

### Study design and participants

In the current research, we employed a cross-sectional design. The participants, identified as people diagnosed with TLE through established clinical protocols, were recruited consecutively. Diagnoses were grounded in seizure semiology and the presence of interictal epileptiform discharges (IEDs) in the electroencephalogram (EEG) indicative of TLE.

The inclusion criteria were patients aged ≥ 18 years, diagnosed with TLE, with a monthly household income below Morocco's guaranteed interprofessional minimum wage (that is, monthly income below US$ 277), who agreed to participate. And the exclusion criteria were patients with concurrent severe neurological and/or psychiatric disorders, illiterate individuals, non-Arabic speakers, subjects experiencing an epileptic seizure within 24 hours preceding the neurocognitive assessment, previous brain surgery recipients, and individuals who refused to participate.

The research was conducted at the Public Health Care Clinic in the city of Agadir, Morocco, a “day clinic” offering neurological consultations to the residents of the Sous Massa region. The study spanned from July 2021 to August 2022.

### Data collection

All patients underwent an extensive anamnesis. The initial data collection included demographic characteristics such as age, sex, level of schooling, occupation, and marital status. Subsequently, clinical details such as familial history of epilepsy, seizure description, age at onset of epilepsy, delays before diagnosis, epilepsy duration, seizure frequency, and treatment were recorded. Furthermore, social behavior support, and cognitive complaints were assessed.

#### *Evaluation of cognitive functions*

Cognitive functioning was assessed using the Montreal Cognitive Assessment (MoCA)—Arabic version. 21 22 The MoCA is recommended as a cognitive screening tool for patients with epilepsy, according to several studies. 23 This instrument covers multiple domains: attention, concentration, executive functions, episodic memory, language, visual constructive praxis, abstraction, calculation, and orientation. Its administration generally takes between 15 and 20 minutes.

#### *Evaluation of anxiety and depression*

The Hospital Anxiety and Depression Scale (HADS)—Arabic version— 24 25 was used to assess anxiety and depression levels. It enables the identification of these disorders while excluding somatic symptoms which could prevent an accurate assessment. Renowned for its efficacy and brevity, the HADS can be administered in 2 to 6 minutes and has proven validity in people with epilepsy. 26

#### *Evaluation of self-esteem*

The Rosenberg Self-Esteem Scale (RSES)—Arabic version— 27 28 was used to assess the participants' self-esteem. The overall score ranges from 10 to 40, with higher scores suggesting higher self-esteem.

#### *Quality of life assessment*

Quality of life was assessed using the Quality of Life Inventory in Epilepsy-31 questionnaire (QOLIE-31)—Arabic version. 29 30 It comprises 31 items assessing 7 domains: Seizure Worry, Overall QoL, Emotional Wellbeing, Energy/Fatigue, Cognitive Functioning, Medication Effects, and Social Functioning.

### Statistical analysis

The normality of the variables studied was examined using the Kolmogorov-Smirnov test. Additionally, Spearman correlations were performed to establish the associations involving the overall QOLIE-31 scores, each of its seven domains, and demographic, clinical, social, psychological, and cognitive variables.

After identifying the factors strongly related to the QoL composite score, linear regression analysis was used to assess the predictive role of independent variables in the five areas (anxiety, depression, seizure frequency, cognitive performance, and self-esteem) of the QoL composite score. Then, we analyzed the QoL domains in relation to these factors separately with linear regression. The level of significance was set at at 5%. Data analyses were conducted using the IBM SPSS Statistics for Windows (IBM Corp., Armonk, NY, United States) software, version 25.0.

## RESULTS

### Sociodemographic and clinical characteristics

A total of 56 participants were interviewed, with a 71.4% response rate ( *n* = 40). Of these, 57.5% were male, and 50% were unemployed. The mean(± standard deviation, SD) age of the participants was of 33.35(± 14.27) years ( Table 1 ).

**Table 1:** Sociodemographic and clinical characteristics of patients with TLE ( *n* = 40)

Characteristics		Mean(± SD)	Median (IQR)
Age (years)		33.35(± 14.27)	29 (17–74)
Age at the onset of epilepsy (years)		15.88(± 12.95)	14.5 (7.25–19)
		**n**	**%**
Sex	Male	23	57.5
Level of schooling	High school/University	21	52.5
Marital status	Married	7	17.5
Occupation	Unemployed	20	50
Family history of epilepsy	Yes	9	22.5
Type of therapy	Polytherapy	16	40
Seizure duration	< 2 minutes	13	32.5
Seizure frequency	Free for ≥ 1 year	12	30
Seizure type	FAS	23	57.5
	FIAS	31	77.5
	FBTCS	26	65
	GS	16	40
	US	2	5

Abbreviations: FAS, focal aware seizure; FBTCS, focal to bilateral tonic-clonic seizure; FIAS, focal impaired awareness seizure; GS, Generalized Seizure; IQR, interquartile range; SD, standard deviation; TLE, temporal lobe epilepsy; US, unknown seizure.

### Overall QoL and scale scores

The mean overall score for QoL was of 48.14(± 22.02). Among the seven domains, the score on Medication Effects was the highest (65.77), and the score on Seizure Worry, the lowest (34.22) ( Table 2 ). The internal reliability coefficient (Cronbach's alpha) of the QOLIE-31 was of 0.9.

**Table 2:** Quality of life of the participants with TLE ( *n* = 40)

	Mean(± SD)	Median (IQR)
**QOLIE31: total score**	48.14(± 22.02)	49.95 (29.77–63.09)
**The QOLIE-31 domain scores**		
Seizure Worry	34.22(± 29.38)	31.32 (10.66–52.99)
Overall Quality of Life	54.63(± 25.63)	50 (45–71.25)
Emotional Wellbeing	51.60(± 22.91)	54 (40–67)
Energy/Fatigue	48.95(± 24.33)	52 ( 27.5–68.75)
Cognitive Functioning	43.53(± 26.48)	42.22 (18.74–62.57)
Medication Effects	65.77(± 34.16)	72.23 ( 42.36–100)
Social Functioning	51.86(± 26.41)	59 ( 27.5–71.5)

Abbreviations: IQR, interquartile range; QOLIE-31, Quality of Life Inventory in Epilepsy-31; SD, standard deviation; TLE, temporal lobe epilepsy.

### Factors associated with the QOL of TLE patients

As seen in Table 3 , there was a strong correlation (r = 0.8; *p* < 0.01) between higher QoL composite scores and higher self-esteem scores. Additionally, the correlation between higher QoL composite scores and strong social support was highly significant (r = 0.538; *p* < 0.01). There were also highly-significant correlations regarding lower QoL composite scores and higher scores for depression (r = -0.729; *p* < 0.01), anxiety (r = -0.696; *p* < 0.01), memory complaints (r = -0.539; *p* < 0.01), and frequency of seizures (r = -0.425; *p* < 0.01). Moreover, the results show negative correlations involving depression, anxiety, cognitive complaints, and seizure frequency and the QOLIE-31 domains. Conversely, self-esteem and social support demonstrated positive correlations with all QOLIE-31 domains, except Medication Effects.

**Table 3:** Correlation analysis of factors influencing the QoL of patients with TLE ( *n* = 40)

	QOLIE-31 total: score	Seizure Worry	Overall Quality of Life	Emotional Wellbeing	Energy/Fatigue	Cognitive Functioning	Medication Effects	Social Function
Age	0.057	0.041	0.155	-0.042	0.069	0.017	0.483**	0.058
evel of schooling	0.096	-0.112	0.131	0.11	0.229	0.003	-0.376*	0.204
Seizure frequency	-0.425**	-0.398*	-0.34*	-0.486**	-0.407**	-0.388*	-0.193	-0.398*
Seizure duration	-0.315*	-0.251	-0.40*	-0.332*	-0.370*	-0.206	-0.109	-0.231
BTCS and GS	-0.341*	0.005	-0.285	-0.397*	-0.472**	-0.259	0.1	-0.36*
Age at seizure onset	-0.009	-0.034	0.038	0.045	0.004	-0.007	-0.07	0.081
Polytherapy	-0.214	-0.139	-0.205	-0.235	-0.171	-0.161	0.255	-0.301
Disease duration	-0.073	-0.181	-0.101	-0.197	-0.074	-0.13	0.315*	-0.089
Depression (HADS-D)	-0.729**	-0.615**	-0.638**	-0.701**	-0.712**	-0.455**	-0.326*	-0.67**
Anxiety (HADS-A)	-0.696**	-0.714**	-0.59**	-0.658**	-0.548**	-0.585**	-0.243	-0.602**
Self-esteem (RSES)	0.800**	0.564**	0.799**	0.766**	0.724**	0.603**	0.243	0.721**
Social support	0.538**	0.51**	0.411**	0.426**	0.284	0.512**	0.108	0.456**
Social Isolation	-0.392*	-0.387*	-0.285	-0.366*	-0.2	-0.239	-0.212	-0.46**
Cognitive complaints	-0.539**	-0.338*	-0.557**	-0.526**	-0.525**	-0.557**	-0.116	-0.491**
Cognitive performance (MoCA)	0.352*	0.137	0.317*	0.327*	0.384*	0.43**	0.075	0.305
Visuospatial/Executive	0.105	-0.155	0.182	0.13	0.191	0.014	-0.343*	0.228
Naming	0.21	-0.101	0.194	0.202	0.271	0.251	-0.313*	0.307
Attention	0.0348*	-00.025	00.303	00.305	00.433**	00.373*	-00.128	00.38*
Language	0.211	-0.054	0.284	0.227	0.353*	0.116	-0.282	0.298
Abstraction	0.222	-0.106	0.213	0.306	0.394*	0.124	-0.079	0.359*
Delayed recall	0.281	0.074	0.334*	0.282	0.337*	0.394*	-0.172	0.268
Orientation	0.141	-0.174	0.115	0.094	0.142	0.139	-0.271	0.290

Abbreviations: BTCS, bilateral tonic-clonic seizure; GS, generalized seizure; HADS-A, Hospital Anxiety and Depression Scale – Anxiety; HADS-D, Hospital Anxiety and Depression Scale – Depression; MoCA, Montreal Cognitive Assessment. QOLIE-31, Quality of Life Inventory in Epilepsy-31; RSES, Rosenberg Self-Esteem Scale; TLE, temporal lobe epilepsy.

Notes: * *p* < 0.05; ** *p* < 0.01.

Furthermore, the correlations involving QoL composite scores and cognitive performance levels (MoCA; r = 0.366; *p* < 0.05), subtest attention (r = 0.348; *p* < 0.05), seizure duration (r = -0.315; *p* < 0.05), bilateral tonic-clonic and generalized seizure (r = -0.341; *p* < 0.05), and social isolation (r = -0.392, *p* < 0.05) were significant. Moreover, there were several statistically-significant correlations, of low to moderate intensity, regarding the QOLIE-31 domains (Energy/Fatigue, Cognitive Functioning, Overall QoL, and Social Functioning) and the MoCA subtests (attention, language, and delayed recall). In addition, the Medication Effects domain was positively correlated with age and negatively correlated with the level of schooling. However, the QoL composite scores did not differ among the groups based on sex, polytherapy, age at epilepsy onset, and disease duration. Therefore, these variables were not included in the posterior regression analysis.

### Regression analysis models for factors influencing the QoL composite score and domains

**Figure 1:**
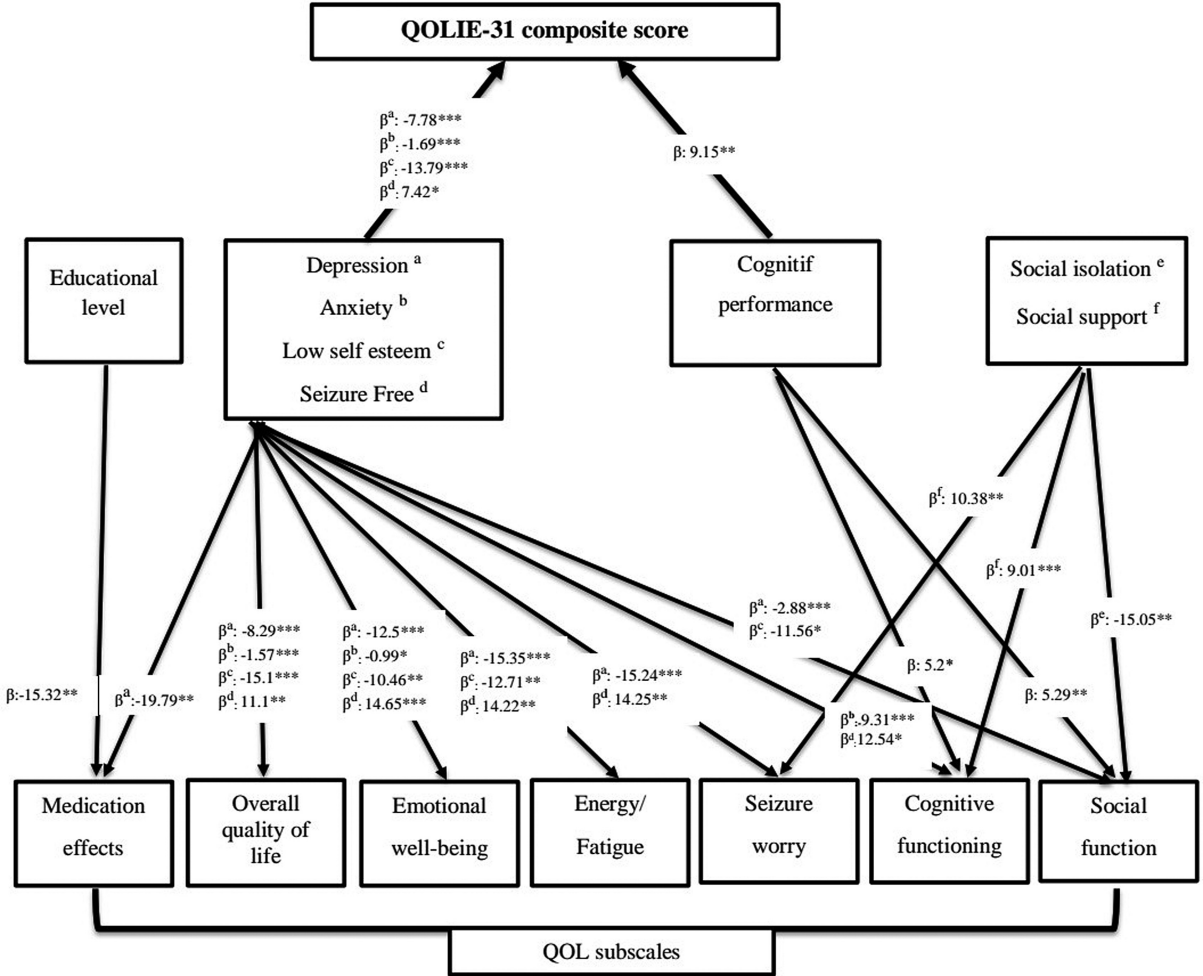
Notes: * *p* < 0.1; ** *p* < 0.05; and *** *p* < 0.001. Summary of the regression analysis models for the factors influencing the quality of life (QoL) composite and domain scores.

A multiple linear regression analysis with stepwise selection better explained the independent associations among the overall QOLIE-31 score and its domains. Five variables were included in the regression model, accounting for 76% of the accumulated variance. This regression model for the QOLIE-31 total score showed a strong r coefficient (0.891; Table 4 and Figure 1 ). In this model, the composite QoL score was predicted by: seizure freedom (β = 7.42; *p* = 0.08), cognitive performance (MoCA score; β = 9.15; *p* = 0.03), depression (β = − 7.78; *p* = 0.00), anxiety (β = − 1.69; *p* = 0.00) and low self-esteem (β = − 13.79; *p* = 0.00).

**Table 4:** Multiple linear regression analysis showing the independent associations involving the clinical, demographic, therapeutic, psychiatric, social, and cognitive variables and the total QOLIE-31 score and the domain scores of patients with TLE

QOLIE-31	r	Adj. r ^2^	β	Std. β	t	*p*
*Total score*	0.891	0.76				
Anxiety			-1.69	-0.4	-3.82	0.00
Seizure free			7.42	0.15	1.77	0.08
Low self-esteem			-13.79	-0.28	-3.31	0.00
Cognitive performance			9.15	0.19	2.23	0.03
Depression			-7.78	-0.3	-2.92	0.00
*Seizure Worry*	0.741	0.511				
Social support			10.38	0.287	2.43	0.02
Anxiety			-15.24	-0.511	-4.28	0.00
Seizure free			14.45	0.22	1.99	0.05
*Overall Quality of Life*	0.86	0.737				
Low self-esteem			-15.1	-0.31	-3.472	0.00
Seizure free			11.1	0.234	2.725	0.01
Anxiety			-1.57	-0.371	-3.389	0.00
Depression			-8.29	-0.318	-2.961	0.00
*Emotional Wellbeing*	0.838	0.669				
Low self-esteem			-10.46	-0.20	-2.06	0.04
Seizure free			14.65	-0.29	3.08	0.00
Anxiety			-0.992	-0.22	-1.82	0.07
Depression			-12.54	-0.46	-3.83	0.00
*Energy/Fatigue*	0.742	0.513				
Seizure free			14.22	0.271	2.37	0.02
Low self-esteem			-12.71	-0.236	-1.95	0.05
Depression			-15.35	-0.533	-4.5	0.00
*Cognitive Functioning*	0.732	0.483				
Social support			9.01	0.368	2.99	0.00
Cognitive performance			5.2	0.21	1.69	0.09
Seizure free			12.54	0.22	1.76	0.08
Anxiety			-9.31	-0.347	-2.8	0.00
*Medication Effects*	0.546	0.26				
Depression			-15.321	-0.379	-2.721	0.01
Level of schooling			-19.796	-0.454	-3.254	0.00
*Social Functioning*	0.836	0.664				
Social isolation			-15.05	-0.24	-2.20	0.03
Low self-esteem			-11.56	-0.2	-1.76	0.08
Depression			-2.88	-0.56	-5.45	0.00
Cognitive performance			5.29	-0.21	2.01	0.05

Abbreviations: Adj., adjusted; QOLIE-31, Quality of Life Inventory in Epilepsy-31; SD, standard deviation; Std. standardized; TLE, temporal lobe epilepsy.

Additionally, several regressions were performed to analyze which QoL domains were more sensitive to factors related to the composite QoL score. Seizure Worry was significantly predicted by anxiety, social support, and seizure freedom. Overall QoL and Emotional Wellbeing were significantly predicted by depression, seizure freedom, anxiety, and self-esteem. All of these factors, except anxiety, were also significant predictors of Energy/Fatigue. Cognitive Functioning was significantly predicted by seizure freedom, social support, anxiety, and cognitive performance, while Medication Effects were predicted by depression and level of schooling. Social Functioning was significantly predicted by social isolation, self-esteem, depression, and cognitive performance.

According to this regression model, the variance in the domains was as follows: 51.1% for Seizure Worry (r = 0.741); 73.7% for Overall QoL (r = 0.86); 66.9% for Emotional Wellbeing (r = 0.838); 51.3% for Energy/Fatigue (r = 0.742); 48.3% for Cognitive Function (r = 0.732); 26% for Medication Effects (r = 0.546) and 66.4% for Social Function (r = 0.836).

### Structural equation modeling (SEM) using Analysis of Moment Structures (AMOS) was performed to identify factors influencing quality of life

**Figure 2:**
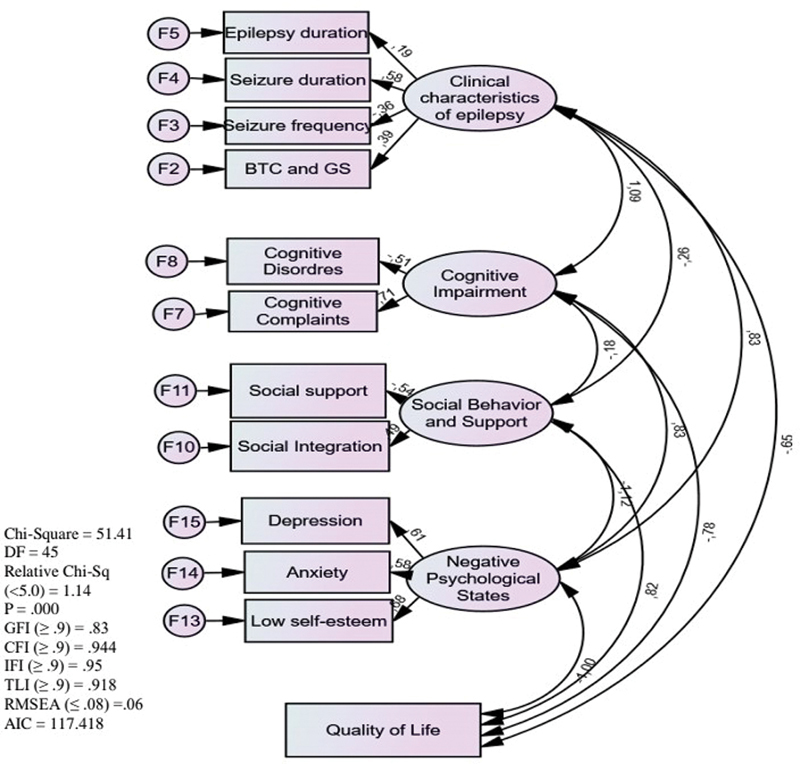
Abbreviations: BTC, bilateral tonic-clonic; GS, generalized seizure; DF, degrees of freedom; QoL, quality of life. Structural equation modelling using the Analysis of Moment Structures (AMOS) software for the factors influencing QoL.

The model in Figure 2 highlights a direct positive association regarding the severity of clinical epilepsy characteristics and cognitive impairment ( *p* = 0.04) and negative psychological states ( *p* = 0.06). This suggests a potential direct influence of epilepsy on an individual's cognitive and psychological wellbeing. Additionally, the model reveals significant negative direct effects of cognitive impairment on social interaction and support ( *p* = 0.00). Similarly, the path coefficient ( *p* = 0.01) indicates a negative association involving negative psychological states and social interaction and support.

Finally, the model demonstrates significant overall negative effects of the severity of clinical epilepsy characteristics, cognitive impairment, and negative psychological states on QoL. In contrast, good social interaction and support positively influence QoL.

## DISCUSSION

Epilepsy research among economically-disadvantaged populations in developing countries is often underrepresented, despite the fact that these individuals face several challenges that can amplify the burden of their illness. The present study aims to address this gap by exploring determinants affecting QoL within these underserved groups. We analyzed multiple dimensions of QoL, including demographic, therapeutic, clinical, psychiatric, social, and cognitive characteristics, and identified the main predictors of QoL among economically-marginalized individuals with TLE.

Our findings revealed a mean QOLIE-31 score of 48.14(± 22.02) points. Notably, this score slightly trails behind the scores observed in TLE patient cohorts from Spain 14 and China, 31 which reported mean QOLIE-31 scores of 52.80(± 13.77) and 50.20(± 15.32) points respectively. However, the score found in the current study was markedly lower than those of samples from Morocco 29 and Nigeria, 12 in which the mean QOLIE-31 scores were of 68(± 22.16) and 77.98(± 13.32) respectively.

Factors such as seizure frequency, duration, bilateral tonic-clonic seizures, and generalized seizures were significantly correlated with the total QOLIE-31 score. These factors were also correlated with various QOLIE-31 domain scores, including Seizure Worry, Overall QoL, Wellbeing, Energy/Fatigue, and Social Functioning. This finding aligns with those of the literature, which indicates that TLE patients with frequent seizures often experience lower QoL. 16 32 Seizure frequency has been identified as a determinant of health-related quality of life (HRQoL) in studies on TLE patients. 33 34 Another study 35 found that generalized seizures were associated with lower scores on the QOLIE-31 domains, such as Seizure Worry, Overall QoL, Emotional Wellbeing, and Cognitive Function. Additionally, seizure severity was inversely correlated with the QOLIE-31 domains.

In the sample of the present study, 22.5% of the participants presented depressive symptoms, while 45% presented signs of anxiety. Anxiety and depression were inversely related to QoL and exhibited profound correlations with all seven QOLIE-31 domains. This evidence underscores the significant role of seizure frequency and severity, along with psychiatric comorbidities, in the decline of QoL among TLE patients—a finding consistently supported by several studies. 14 31 36

A substantial proportion of our participants (72.5%) presented low self-esteem, whose metrics were strongly correlated with the overall QoL across most domains. Although the existing literature 37 suggests a gradual erosion of self-esteem among individuals with epilepsy, there is a gap in academic research on the complex interplay between self-esteem and QoL. One study 38 found that experiences of self-conscious emotions (SCEs) could be dysregulated in individuals with TLE, with negative SCEs (such as shame and guilt) associated with lower QoL. Misconceptions linking epilepsy to supernatural phenomena can lead to societal stigmatization and discrimination, which, in turn, reduces self-esteem. 39 40 41

Regarding cognitive functions, 67.5% of the participants reported cognitive complaints, with 70% presented broader cognitive deficits. Our data revealed strong correlations involving the MoCA scores and the overall QOLIE-31 score, particularly within the Cognitive Functioning domain. Some studies highlight cognitive performance as a crucial determinant of QoL, though other studies present contradictory evidence. 14 16 17 31 Impaired cognitive functions (attention, memory, and language) significantly affect various aspects of QoL. Another study 42 found that language, memory, and attention are key neuropsychological predictors of QoL. Additionally, cognitive complaints are frequently reported by epilepsy patients as significant issues. 43 These findings support the view that a QoL model excluding cognitive functioning is incomplete, emphasizing the importance of both “objective” and “subjective” assessments of cognition in QoL research. 42

It is noteworthy that QoL domains present varying correlations with the studied variables, such as cognitive performance. Specifically, the Cognitive and Social Functioning domains were most influenced by cognitive performance scores. This is in line with the study by Lozano-García et al. (2021). 14 Therefore, comprehensive assessment and management of cognitive dysfunction are crucial components of care for TLE patients.

Our findings also show that social isolation negatively impacts QoL, while adequate social support improves it. Other studies have confirmed that individuals with epilepsy may experience social isolation due to restrictions, stigma, and other factors, which affects their employment, and their marital family lives. Conversely, strong social support can enhance self-efficacy and QoL among people with epilepsy. 44 45

In the sample of the current study, two sociodemographic characteristics were associated with the QoL Medication Effects domain, which was positively correlated with age and negatively correlated with the level of schooling. Older patients might be better adapted to medication regimens, while those with higher levels of schooling are more aware of medication side effects and express greater concern about them. Although the number of antiepileptic drugs (ASMs) is often reported as a negative predictor of QoL, 31 36 it showed no correlation with the total QoL or domain scores in this group. This discrepancy may be due to the small sample size, and further research is needed to explore this issue.

The multivariate regression analysis revealed that anxiety, depression, seizures, cognitive performance, and self-esteem accounted for ∼ 76% of the variance in the overall QOLIE-31 score among low-income TLE patients. Literature reviews have reported several multivariate regression models with different results based on specific variables and contexts. For instance, Chen et al. 31 (2018) found that anxiety, prolonged seizure duration, ASM side effects, and depression explained ∼ 60.6% of the variance in the overall QOLIE-31 score. Conversely, Pauli et al. 46 (2012) identified history of initial precipitant injury, family history of epilepsy, disease duration, age at epilepsy onset, seizure frequency, and psychiatric diagnosis as explaining 36% of the variance in the QOLIE-31 score.

Other studies have identified varying predictors of QoL in TLE patients. Lozano-García et al. 14 (2021) showed that, while cognitive performance was a significant predictor of QoL, emotional variables (trait anxiety and depression) were the most powerful predictors. Similarly, Johnstone et al. 33 (2021) proposed that psychiatric symptomatology, depression, and cognition were stronger determinants of QoL than seizure frequency. Ehrlich et al. 36 (2019) found that depressive symptoms were the most significant predictor of the total QOLIE score, explaining 43.4% of the variance, with executive functioning accounting for an additional 5%. Conversely, Cano-López et al. 47 (2018) found that the QoL composite score was predicted by lowest trait anxiety, lowest depression, lowest neurosensory symptoms, and highest long-term verbal memory with semantic cues.

In conclusion, the present study provides valuable insights into the complex interplay involving clinical epilepsy variables, social behavior and support, psychological states, cognitive function, and QoL. The application of structural equation modeling (SEM) with the Analysis of Moment Structures (AMOS) software enabled a comprehensive examination of these relationships, revealing significant interactions that highlight the multifaceted nature of epilepsy and its impact on the wellbeing of individuals. While freedom from seizures remains a crucial treatment goal for TLE, it is important to consider psychosocial factors for intervention programs and clinical research designed to improve QoL. Psychotherapy and psychopharmacological treatments, as well as cognitive remediation interventions, appear to be effective methods to improve cognitive functioning and overall QoL. Enhancing self-worth, developing effective coping strategies, and integrating social workers and psychologists into routine care can further improve QoL. 39 48 49 50

### Limitations and recommendations

These results should be considered with an awareness of their inherent limitations. Specifically, the reliance on self-reported data may introduce potential biases. The limited sample size constrains the ability to generalize findings to larger populations and restricts complex stratifications for in-depth analyses. Larger and more diverse cohorts could provide more comprehensive information and enhance statistical robustness. Therefore, further research with expanded and varied samples is essential to validate and extend these findings.

In conclusion, the present study revealed that patients with TLE and limited resources experience reduced QoL across all domains and overall scores. Notably, the Seizure Worry domain was found to be the most affected. The findings indicate that higher scores in cognitive functions, social support, and self-esteem are positively associated with QoL. Conversely, cognitive complaints, seizure duration, seizure frequency, bilateral tonic-clonic and generalized seizures, social isolation, anxiety, and depression were negatively associated with QoL. Comprehensive therapies and interventions that address comorbidities and cognitive deficits are crucial to improve QoL.
